# Phytoplankton community and physical-chemical data measured in the Gulf of Trieste (northern Adriatic Sea) over the period March 2006–February 2007

**DOI:** 10.1016/j.dib.2018.05.054

**Published:** 2018-05-17

**Authors:** Tamara Cibic, Cinzia Comici, Claus Falconi, Daniela Fornasaro, Ana Karuza, Marina Lipizer

**Affiliations:** Istituto Nazionale di Oceanografia e di Geofisica Sperimentale - OGS, Sezione di Oceanografia, Via A. Piccard, 54, 34151 Trieste, Italy

## Abstract

Biological, hydrological and chemical data were acquired at monthly intervals from March 2006 to February 2007, at the Long-Term Ecological Research site C1 in the Gulf of Trieste, in the northernmost part of the Adriatic Sea. The biological dataset comprises total chl *a* and phaeopigment concentrations, and the distinction of the total phytoplankton biomass into three photoautotrophic community fractions, i.e. cyanobacteria, nano- and microphytoplankton, collected at discrete depths. Hydrological data encompass the thermohaline properties of the water column (temperature and salinity profiles from CTD casts). Chemical data consist of silicate and phosphate concentrations obtained from discrete seawater samples collected with Niskin bottles at four depths (0.5–5–10–15 m). Data presented here are related to the paper “Structural and functional response of phytoplankton to reduced river inputs and anomalous physical-chemical conditions in the Gulf of Trieste (northern Adriatic Sea) by Cibic et al. (2018) [1].

**Specifications Table**

**Table:** 

Subject area	*Marine ecology*
More specific subject area	*Physical-, chemical- and biological oceanography*
Type of data	*Table and figure*
How data was acquired	*Temperature and salinity: CTD probe SBE 19plus SeaCAT profiler (Sea-Bird Electronics); cyanobacteria and nanophytoplankton: epifluorescence microscopy (Olympus BX51*); *microphytoplankton: inverted light microscopy (Leitz Fluovert FS); inorganic nutrients: spectrophotometric method (Bran-Luebbe Autoanalyzer 3); pigments: spectrofluorometric method (PerkinElmer LS-50B).*
Data format	*Averaged, analyzed, processed*
Experimental factors	*From March 2006 to February 2007, discrete seawater samples were collected monthly with 5-L Niskin bottles at four depths (0.5, 5, 10, 15 m) for inorganic nutrient, phytoplankton and pigment analyses. During sampling, seawater temperature and salinity were recorded by a CTD probe.*
Experimental features	*Link the phytoplankton community biomass and structure to thermohaline features and inorganic nutrient availability in the Gulf of Trieste.*
Data source location	*Trieste, Italy, Long-Term Ecological Research (LTER) site C1 (45°42′2′′N and 13°42′36′′E)*
Data accessibility	*Data are presented in this article*
Related research article	*Cibic et al.*[1]

**Value of the data**

• Data provide information on the phytoplankton biomass, in terms of chl *a*, and its division into three photoautotrophic communities of different size-classes.
• Data on cyanobacteria, nanophyto- and microphytoplankton, obtained synoptically, may be used as a baseline for future studies.
• Data on temperature and salinity highlight anomalous thermohaline features in a shallow basin and may be used for future comparisons with similar temperate semi-enclosed seas.
• Inorganic nutrient data, highlighting silicate depletion for diatom growth, could be valuable to researchers investigating coastal oligotrophic ecosystems.
• Data here presented may be used to study the effects of environmental factors on phytoplankton biomass and community structure.

## 1 Data

The biomass of three different size-classes of phytoplankton, i.e. cyanobacteria (0.2–2 µm), nano- (2–20 µm) and microphytoplankton (20–200 µm), expressed as percentage of the total phytoplankton, is presented in Table 1. Along the water column, cyanobacteria were the prevalent phototrophs in late summer-early autumn (September and October). The nanophytoplankton reached the highest fractions of the phytoplankton biomass in March 2006 and January 2007, whereas the large-size (> 20 µm) phytoplankton cells dominated in summer, May and November.

**Table 1:** Cyanobacteria (cyano), nano- (nano) and microphytoplankton (micro) fractions of total phytoplankton biomass at each depth from March 2006 to February 2007. Data, obtained from abundance values, were converted into biomass (for conversion factors applied to the three phytoplankton communities see Section 2), and expressed as percentage.

	**0.5 m**			**5 m**			**10 m**			**15 m**		
	**Cyano**	**Nano**	**Micro**	**Cyano**	**Nano**	**Micro**	**Cyano**	**Nano**	**Micro**	**Cyano**	**Nano**	**Micro**
**Mar 06**	7.4	74.0	18.6	6.4	82.5	11.1	5.2	89.8	5.0	10.4	83.5	6.1
**Apr**	36.7	49.4	13.9	31.3	38.3	30.3	31.0	68.7	0.3	38.1	54.0	7.9
**May**	1.1	13.1	85.8	1.2	15.0	83.8	0.4	10.9	88.6	0.3	10.0	89.7
**Jun**	4.2	44.4	51.4	2.5	60.9	36.6	3.2	49.4	47.4	3.2	42.7	54.1
**Jul**	1.8	3.0	95.2	1.4	2.5	96.1	1.2	1.7	97.1	0.7	3.0	96.2
**Aug**	7.4	17.5	75.1	9.9	32.1	58.0	7.0	17.3	75.8	7.8	17.2	75.0
**Sep**	64.2	32.9	2.9	63.3	23.8	12.8	61.2	20.4	18.4	44.0	27.8	28.2
**Oct**	35.5	34.2	30.3	32.0	27.2	40.8	27.5	13.3	59.2	23.5	27.9	48.6
**Nov**	4.8	20.3	74.9	1.9	28.5	69.6	3.8	24.5	71.8	9.3	37.4	53.3
**Dec**	11.8	33.9	54.3	8.8	53.9	37.2	8.0	87.7	4.3	11.3	84.5	4.2
**Jan 07**	1.8	88.7	9.5	1.5	78.0	20.5	2.1	86.6	11.3	1.6	82.3	16.1
**Feb**	0.5	39.8	59.7	0.4	36.8	62.9	0.4	37.0	62.6	0.3	29.1	70.6

**Fig. 1:**
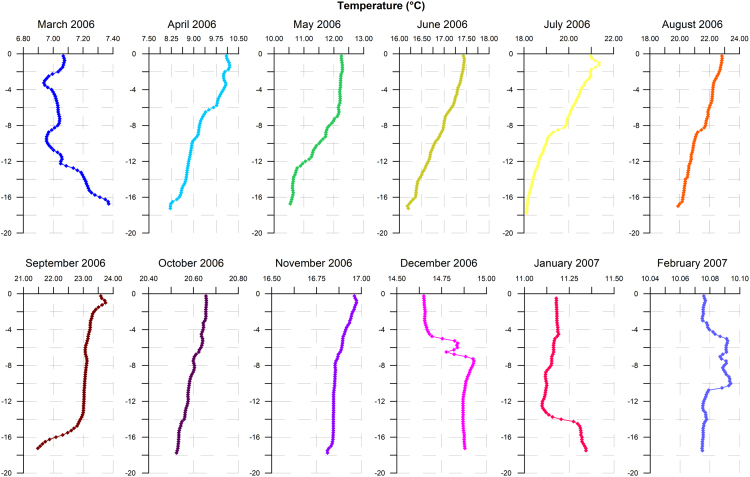
Temperature profiles recorded along the water column in the study site during monthly samplings carried out from March 2006 to February 2007.

**Fig. 2:**
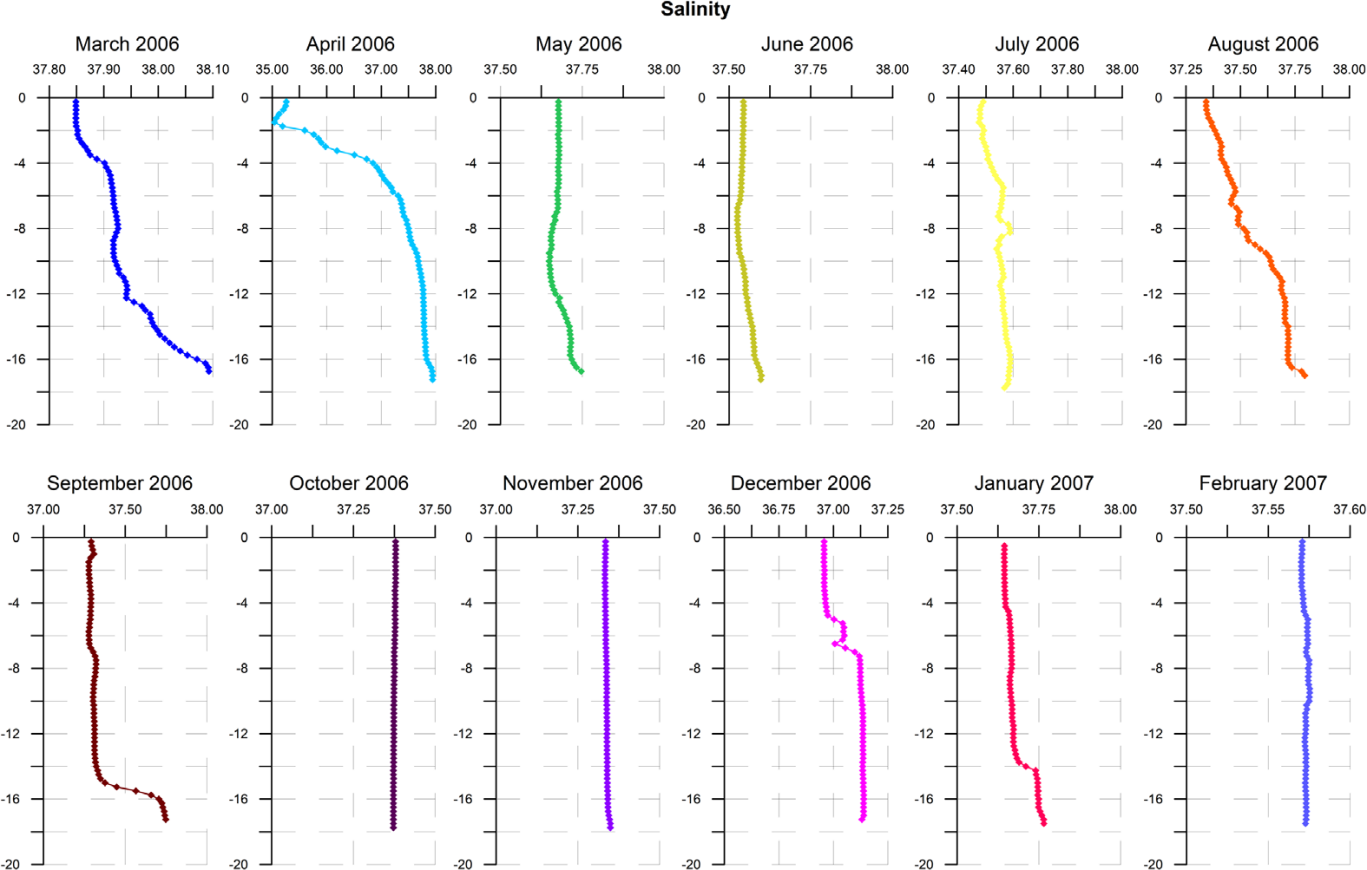
Salinity profiles recorded along the water column in the study site during monthly samplings carried out from March 2006 to February 2007.

Temperature profiles recorded along the water column are shown in Fig. 1. Comparable temperatures were registered in June and November 2006 along the water column, as well as in April 2006 and February 2007 at the uppermost 3-m-layer. Salinity profiles are shown in Fig. 2. At the surface layer, the highest salinity was recorded in March 2006 while the lowest one was registered in the following month.

Table 2 presents phosphate and silicate concentrations measured at the four discrete depths, and their ratios. Silicate limitation was recorded in May and July 2006, and February 2007. Chl *a* and phaeopigment (phaeo) concentrations, and their ratios, analysed at the four sampling depths over the study year, are listed in Table 3. The highest chl *a* concentrations were reached in November 2006 whereas major phaeo/chl *a* ratios were obtained in December 2006 and January 2007, particularly at the bottom layers.

**Table 2:** Phosphate and silicate concentrations at the four depths and their ratios. Si/P < 15 indicates Si limitation [3].

Sampling	Depth	P-PO_4_	Si-Si(OH)_4_	Si/P	Limitation
dd/mm/yyyy	m	µM	µM		
08/03/2006	0.5	0.03	1.64	54.7	
	5	0.04	2.10	52.5	
	10	0.04	2.20	55.0	
	15	0.05	2.34	46.8	
05/04/2006	0.5	0.05	6.80	136.0	
	5	0.05	4.03	80.6	
	10	0.04	3.54	88.5	
	15	0.06	4.03	67.2	
04/05/2006	0.5	0.10	1.44	14.4	Si limitation
	5	0.07	0.87	12.4	Si limitation
	10	0.10	0.48	4.8	Si limitation
	15	0.07	1.45	20.7	
07/06/2006	0.5	0.02	1.26	63.0	
	5	0.06	0.92	15.3	
	10	0.06	1.51	25.2	
	15	0.07	1.2	17.1	
06/07/2006	0.5	0.04	0.94	22.7	
	5	0.03	0.36	12.1	Si limitation
	10	0.04	0.53	13.3	Si limitation
	15	0.04	0.29	7.3	Si limitation
08/08/2006	0.5	0.05	1.86	40.8	
	5	0.03	1.76	58.6	
	10	0.11	2.42	22.0	
	15	0.05	2.33	46.6	
05/09/2006	0.5	0.01	1.25	115.1	
	5	0.01	1.63	163.4	
	10	0.01	1.16	116.0	
	15	0.01	3.34	334.0	
10/10/2006	0.5	0.02	3.71	150.0	
	5	0.03	5.92	197.4	
	10	0.06	5.93	98.8	
	15	0.06	5.19	86.5	
08/11/2006	0.5	0.03	3.18	119.0	
	5	0.05	2.37	47.4	
	10	0.02	2.76	138.0	
	15	0.01	3.04	304.0	
05/12/2006	0.5	0.09	4.63	52.6	
	5	0.08	2.92	36.5	
	10	0.12	4.44	37.0	
	15	0.09	4.12	45.8	
10/01/2007	0.5	0.04	2.47	61.8	
	5	0.07	3.86	55.1	
	10	0.02	2.16	108.0	
	15	0.09	2.30	25.6	
06/02/2007	0.5	0.10	1.03	10.3	Si limitation
	5	0.07	1.03	14.8	Si limitation
	10	0.06	1.04	17.3	
	15	0.10	1.68	16.8

**Table 3:** Chlorophyll *a* (chl *a*) and phaeopigment (phaeo) concentrations, expressed as µg L^−1^, and their ratios, at the four sampling depths during the study period.

**Sampling**	**Depth**	**chl*****a***	**phaeo**	**phaeo/chl*****a***
dd/mm/yyyy	m	µg L^−1^	µg L^−1^	
08/03/2006	0.5	0.56	0.07	0.13
	5	0.55	0.00	0.00
	10	0.51	0.08	0.16
	15	0.61	0.16	0.26
05/04/2006	0.5	0.63	0.45	0.71
	5	0.77	0.60	0.78
	10	0.77	0.69	0.90
	15	0.78	0.63	0.81
04/05/2006	0.5	0.89	0.46	0.51
	5	0.47	0.30	0.63
	10	0.37	0.25	0.67
	15	0.75	0.53	0.70
07/06/2006	0.5	0.49	0.72	1.48
	5	0.76	1.13	1.48
	10	1.29	2.30	1.79
	15	1.70	3.68	2.16
06/07/2006	0.5	0.34	0.23	0.68
	5	0.44	0.19	0.42
	10	1.15	0.74	0.64
	15	2.35	1.79	0.76
08/08/2006	0.5	0.33	0.17	0.51
	5	0.29	0.20	0.67
	10	0.54	0.71	1.31
	15	1.34	2.04	1.52
05/09/2006	0.5	0.62	0.47	0.77
	5	0.42	0.54	1.31
	10	0.42	0.54	1.29
	15	1.83	1.75	0.96
10/10/2006	0.5	0.52	0.95	1.81
	5	0.43	0.80	1.85
	10	0.48	0.74	1.55
	15	0.26	0.49	1.85
08/11/2006	0.5	1.24	1.16	0.93
	5	3.69	3.90	1.06
	10	2.70	2.86	1.06
	15	1.18	1.20	1.01
05/12/2006	0.5	0.63	1.84	2.90
	5	0.39	0.80	2.06
	10	0.20	0.84	4.12
	15	0.08	0.32	4.05
10/01/2007	0.5	0.58	1.96	3.40
	5	0.56	1.75	3.11
	10	2.10	1.66	0.79
	15	0.47	2.08	4.43
06/02/2007	0.5	1.71	1.32	0.77
	5	2.10	1.55	0.74
	10	2.02	1.41	0.70
	15	1.80	1.99	1.10

## 2 Experimental design, materials, and methods

### 2.1 Sampling and environmental data collection

Sampling was performed monthly at the Long-Term Ecological Research (LTER) station C1 (45°42′2′′N and 13°42′36′′E, maximum depth 17.5 m) located in the Gulf of Trieste, in the northernmost part of the Adriatic Sea. From March 2006 to February 2007, vertical profiles of seawater temperature and salinity were recorded by a CTD probe model Sea-Bird Electronics SBE 19plus SeaCAT profiler. Discrete seawater samples were collected monthly with 5-L Niskin bottles at four depths (0.5, 5, 10, 15 m) for inorganic nutrient, pigment and phytoplankton analyses.

### 2.2 Inorganic nutrient and pigment analyses

Samples for the determination of dissolved inorganic nutrient (phosphate, P-PO_4_; and silicate, Si-Si(OH)_4_) concentrations were prefiltered through 0.7 µm pore size glass-fibre filters (Whatmann GF/F), stored at − 20 °C and analysed by a flow injection spectrophotometric method on a five-channel Bran-Luebbe Autoanalyzer 3 using standard procedures [2]. To highlight silicate limitation, the Redfield-Brzezinski nutrient ratio of C:Si:N:P = 106:15:16:1 for diatoms was applied to our dataset [3].

Subsamples for chl *a* analysis were stored in the dark and kept at 4 °C until filtration through 47 mm Whatman GF/F filters that were then stored frozen (− 20 °C) until laboratory analysis. Pigments were extracted overnight (4 °C) with 90% acetone and determined spectrofluorometrically [4]. The measurements of chl *a* and phaeopigments were performed, respectively, before and after acidification with two drops of HCl 1N using a PERKIN ELMER LS-50B spectrofluorometer.

### 2.3 Determination of different phytoplankton size-classes

The cyanobacteria (0.2–2 µm) abundance was estimated from 50 mL-samples, preserved in 0.2 µm pre-filtered formaldehyde (2% v/v final concentration) in the dark at 4 °C and processed within 48 h. Samples were filtered in triplicate (3–15 mL per subsample) through 0.2 µm pore-size black-stained polycarbonate membranes (Ø 25 mm, Nuclepore). Filters were mounted on microscope slides using non-fluorescent oil and stored at − 20 °C. The enumeration was carried out using an Olympus BX51 epifluorescence microscope equipped with a 100 W high-pressure mercury burner (HPO 100 W/2) at 1000× final magnification. Cells were counted in randomly selected fields under green (BP 480–550, BA 590 nm) filter set. A minimum of 200 cells was accounted for each sample. Cyanobacteria cell numbers were converted into carbon biomass using a factor of 200 fg C cell^−1^ [5].

For nanophytoplankton (2–20 µm) analysis, water samples were collected in 100 mL-dark bottles, fixed with prefiltered glutaraldehyde (1% final concentration) and stored in the dark at 4 °C until the analyses. Subsamples (30 mL) were filtered at low pressure (max 100 mmHg) through 0.8 mm pore-size black polycarbonate membranes (Ø 25 mm, Nuclepore). Filters were stained with DAPI (4′6′-diamidino-2-phenylindole) and mounted on glass slides in three replicates for each sample [6]. A minimum of 200 nanophytoplankton cells per filter were counted in randomly selected fields at a 1000× final magnification using an Olympus BX51 microscope equipped with a mercury burner light. The set of filters for chlorophyll fluorescence (BP450–490/FT 510/LP520) was used. The biovolume was estimated and converted to carbon content using a conversion factor of 0.14 pg C µm^−3^ [7].

For microphytoplankton (20–200 µm) analysis, samples were collected in 500 mL-dark bottles and preserved with prefiltered and neutralized 1.6% formaldehyde [8]. Cell counts of the microphytoplankton were performed following the Utermöhl method [9]. A variable volume of seawater (25–50 mL) was settled depending on cell concentrations. Counting was performed in random fields (20–40) using an inverted microscope (Leitz Fluovert FS) equipped with phase contrast, at a final magnification of 320×. In addition, one half of the Utermöhl chamber was also examined at a magnification of 200×, to obtain a more correct evaluation of less abundant microphytoplankton taxa. The biovolume of the microphytoplankton cells was calculated from cell-size and shape by using appropriate geometric formulas [10], [11]. Cell volumes were then converted to carbon content using the formula introduced by Menden-Deuer and Lessard [12].
